# Comparative analysis reveals unexpected genome features of newly isolated Thraustochytrids strains: on ecological function and PUFAs biosynthesis

**DOI:** 10.1186/s12864-018-4904-6

**Published:** 2018-07-17

**Authors:** Zhiquan Song, Jason E. Stajich, Yunxuan Xie, Xianhua Liu, Yaodong He, Jinfeng Chen, Glenn R. Hicks, Guangyi Wang

**Affiliations:** 1 Center for Marine Environmental Ecology, School of Environmental Science and Engineering, Tianjin University, Tianjin, 300072 China; 2 Key Laboratory of Systems Bioengineering (Ministry of Education), Tianjin University, Tianjin, 300072 China; 3 Department of Plant Pathology and Microbiology, University of California, Riverside, California 92521 USA; 4 Department of Botany and Plant Sciences, University of California, Riverside, California 92521 USA; 5 Institute for Integrative Genome Biology, University of California, Riverside, California 92521 USA

**Keywords:** Thraustochytrids, Whole genome sequencing, Polyunsaturated fatty acids, Comparative genomics, Ecological function

## Abstract

**Background:**

Thraustochytrids are unicellular fungal-like marine protists with ubiquitous existence in marine environments. They are well-known for their ability to produce high-valued omega-3 polyunsaturated fatty acids (ω-3-PUFAs) (e.g., docosahexaenoic acid (DHA)) and hydrolytic enzymes. Thraustochytrid biomass has been estimated to surpass that of bacterioplankton in both coastal and oceanic waters indicating they have an important role in microbial food-web. Nevertheless, the molecular pathway and regulatory network for PUFAs production and the molecular mechanisms underlying ecological functions of thraustochytrids remain largely unknown.

**Results:**

The genomes of two thraustochytrids strains (Mn4 and SW8) with ability to produce DHA were sequenced and assembled with a hybrid sequencing approach utilizing Illumina short paired-end reads and Pacific Biosciences long reads to generate a highly accurate genome assembly. Phylogenomic and comparative genomic analyses found that DHA-producing thraustochytrid strains were highly similar and possessed similar gene content. Analysis of the conventional fatty acid synthesis (FAS) and the polyketide synthase (PKS) systems for PUFAs production only detected incomplete and fragmentary pathways in the genome of these two strains. Surprisingly, secreted carbohydrate active enzymes (CAZymes) were found to be significantly depleted in the genomes of these 2 strains as compared to other sequenced relatives. Furthermore, these two strains possess an expanded gene repertoire for signal transduction and self-propelled movement, which could be important for their adaptations to dynamic marine environments.

**Conclusions:**

Our results demonstrate the possibility of a third PUFAs synthesis pathway besides previously described FAS and PKS pathways encoded in the genome of these two thraustochytrid strains. Moreover, lack of a complete set of hydrolytic enzymatic machinery for degrading plant-derived organic materials suggests that these two DHA-producing strains play an important role as a nutritional source rather than a nutrient-producer in marine microbial-food web. Results of this study suggest the existence of two types of saprobic thraustochytrids in the world’s ocean. The first group, which does not produce cellulosic enzymes and live as ‘left-over’ scavenger of bacterioplankton, serves as a dietary source for the plankton of higher trophic levels and the other possesses capacity to live on detrital organic matters in the marine ecosystems.

**Electronic supplementary material:**

The online version of this article (10.1186/s12864-018-4904-6) contains supplementary material, which is available to authorized users.

## Background

Heterotrophic microbes are a key element in microbial food web to control both material and energy flow in the world’s oceans. Thraustochytrids are unicellular fungal-like marine protists found ubiquitously in marine environments [1] and have long been thought to play significant role in marine microbial ecology. Their metabolic processes include utilization of autochthonous particulate organic carbon (POC) as nutritional carbon sources [2]. They have been ascribed a wide range of abilities to reside on multiple substrates with their own spatial and trophic niches in the ocean [3]. The biosynthetic capabilities of thraustochytrids have been exploited for broad biotechnological applications as they can produce an array of hydrolytic enzymes for highly refractory organic plant matter, which is difficult for most marine bacterioplankton to digest, suggesting distinct ecological roles that differ from their prokaryotic counterparts [4, 5]. The biomass of thraustochytrids is reported to greatly exceed that of bacterioplankton in coastal and oceanic waters supporting their perhaps underappreciated importance in microbial food web and ocean carbon cycling [6, 7]. Finally, many strains of thraustochytrids grow quickly and accumulate high levels of omega-3 polyunsaturated fatty acids (ω-3 PUFAs) (docosahexaenoic acid (DHA) and eicosapentaenoic acid (EPA)), which are important materials in the flow of energy in marine ecosystems and also high-valued nutraceuticals [8, 9]. However, neither the metabolic pathways for the production of these lipids and enzymes nor the regulatory network for their production is known.

An initial collection of thraustochytrids genomic resources has been developed, including a draft genome, transcriptome and gene expression analyses of the thraustochytrid protistan parasite (Quahog Parasite Unknown, QPX). Analyses of these data have provided insight into the causative agent of large-scale mortalities in hatchery-reared and commercially harvested hard clams (quahogs; *Mercenaria mercenaria*) in the northeastern coast of North America [10]. The 34.7 Mb genome sequence of this pathogen has provided some key information to improve our understanding of the molecular mechanisms underlying the physiological responses to temperature associated stress for this temperature-dependent pathogen. Draft genome sequences of two DHA-producing thraustochytrid strains *Schizochytrium* sp. (CCTCC M209059) [11] and *Aurantiochytrium* sp. strain T66 [12] have been produced along with three additional thraustochytrids, *A. limacinum* (ATCC MYA-1381), *A. kerguelense* (PBS07) and *S. aggregatum* (ATCC 28209) by the US Department of Energy, Joint Genome Institute (http://genome.jgi.doe.gov/portal/). The strains *Schizochytrium* sp. CCTCC M209059, *Aurantiochytrium* sp. strain T66, and *A. limacinum* ATCC MYA-1381 have been reported be high producers of DHA [11, 13, 14]. However, a systematic analysis of molecular synthesis and regulatory networks for PUFAs and hydrolytic enzyme biosynthesis has not been performed to compare and contrast the evolution and stability of these pathways. In addition, the molecular mechanisms underlying ecological functions of thraustochytrids have yet to be explored using genomic information.

In this study, we report high-quality genome assembly of two PUFAs producing thraustochytrid strains isolated from marine habitats in the coastal water of Southern China. Their detailed genomic maps were constructed using the second generation and single-molecule sequencing data and improved with information derived RNA-seq data. The comparative genomic analyses provide new insight into the evolution and variation in PUFAs biosynthesis and molecular machineries of their functional ecology of thraustochytrids. Our work represents a comprehensive analysis of thraustochytrid genomes laying a framework for future molecular ecology study and biotechnological utility of these thraustochytrids strains.

## Results

### Genome sequencing and assembly

The genomes of two thraustochytrid strains *Schizochytrium* sp. (Mn4) and *Thraustochytriidae* sp. (SW8) were sequenced at 60× coverage using Illumina HiSeq 2500 (40×) and single-molecule real-time sequencing analysis PacBio RS (20×). Quality control assessment of the Illumina sequencing reads found a Q20 (rate of sequencing errors less than 1%) for Mn4 of 98.42% and for SW8 a rate of 98.58% (Additional file 1: Table S1). The GC ratio of sequence reads for both of these thraustochytrid strains was on average 45%. After filtering, the PacBio subreads lengths N50 (including adaptors) for Mn4 and SW8 was 17,462 and 17,769 (Additional file 1: Table S1). Finally, the hybrid assembled genome of Mn4 had a total length of 65.69 Mb and N50 of 153 kb (57× effective sequence depth and 99.95% coverage) and total size of 61.67 Mb and N50 of 127 kb for SW8 (60× and 99.96% coverage) (Additional file 1: Table S2). There were 1161 scaffolds greater than 1 Kb for Mn4 and 1202 for the SW8 assembly. BUSCO [15] analysis for the assessment of annotated gene sets of genomes indicated the hybrid assemblies are of better completeness (Mn4: 91.40%; SW8: 91.80%) than the HiSeq alone (Mn4: 87.80%; SW8: 87.80%) (Additional file 1: Table S2).

### Genome sequence annotation

The gene annotation identified 17,887 and 16,574 protein-coding genes in the genomes of the strain Mn4 and SW8, respectively. A total of six genomes of thraustochytrids were used for comparative analysis. Of these six genomes, the strains Mn4, SW8, CCTCC M209059 and ATCC MYA-1381 have the ability to produce DHA. According to the genome statistics, three DHA-producing strains, Mn4, SW8 and *A. limacinum* ATCC MYA-1381 had relatively high-quality assembled genomes whose size ranged from 58.10 to 65.69 Mb (Table 1). Comparing genome size of the three DHA-producing strains and the other two non DHA-producing strains (the strains PBS07 and ATCC 28209), the genomes of DHA-producing strains were at least 48.72% larger than those of the non DHA-producing strains.

**Table 1:** Thraustochytrids genome statistics

Characteristic	Mn4^a^	SW8^a^	*Schizochytrium* sp. CCTCC M209059^a^	*A. limacinum* ATCC MYA-1381^a^	*A. kerguelense* PBS07	*S. aggregatum* ATCC 28209
Gene ID prefix	Mn4	SW8	SchiM	Aurli1	Aplke1	Schag1
scaffolds	1611	1202	322	181	207	283
assembled genome (Mb)	65.69	61.67	37.28	58.10	34.12	38.96
rate of N	0%	0%	2%	2%	4%	5%
rate of GC	45%	45%	57%	45%	41%	63%
scaffold N50 (kp)	154	128	596	2464	718	635
scaffold N90 (bp)	14,564	22,659	144,465	790,046	313,860	162,527
sequences> = 1 kb	1611	1202	322	181	207	283
sequences> = 2 kb	1583	1189	247	181	130	163
sequences> = 3 kb	1509	1149	218	89	126	156
No. of predicted CDS	17,887	16,574	12,407	14,859	11,892	10,612
No. of secreted proteins	297	252	330	324	316	297

^a^The DHA-producing thraustochytrids strains

**Fig. 1:**
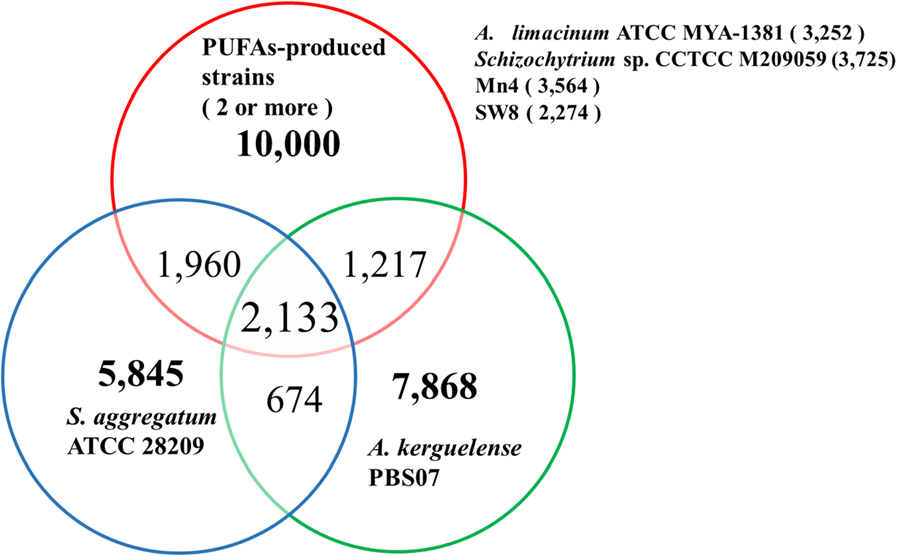
Common and unique thraustochytrids ortholog groups. This Venn diagram shows unique and overlapping gene families in the PUFAs-producing strains and two non PUFAs-producing thraustochytrids (*Schizochytrium aggregatum* ATCC 28209 and *Aplanochytrium kerguelense* PBS07)

Orthology analysis indicated shared gene structure and functional genic information among four DHA-producing strains. All four DHA-producing strains shared 6625 orthologous groups, which encompassed from 37% (Mn4) to 53% (strain CCTCC M209059) of the total gene set in their individual genomes. After excluding these unique genes, 15,310 genes were found in two or more genomes of DHA producing strains and were combined into one set for comparing with those in the individual genomes of the two non DHA-producing strains (PBS07 and ATCC 28209). The comparison revealed that 2133 core orthologous groups were shared among the genomes of four DHA-producing and two non DHA-producing strains (Fig. 1). Still, a total of 3177 genes were identified to be shared with those in either genome of PBS07 and ATCC 28209. Thus, 10,000 orthologous groups were unique the DHA-producing gene set. Furthermore, of these unique orthologous groups, 2778 were found in the genomes of all four DHA-producing strains. Thus, this gene set contains the pathways of genes utilized for DHA-production by these strains.

### Gene ontology analysis

Although Mn4 and SW8 were isolated from different marine habitats, little difference was observed in the predicted proteome when comparing GO categories for cellular component, molecular function, and biological processes (Additional file 2: Figure S1). Evaluation of abundance or depletion of GO terms assignments of genes found the abundant pigmentation (GO:0043473) biological process was enriched in the DHA-producing strains which is consistent with previous reports of high carotenoid production capacity by thraustochytrids [16].

**Fig. 2:**
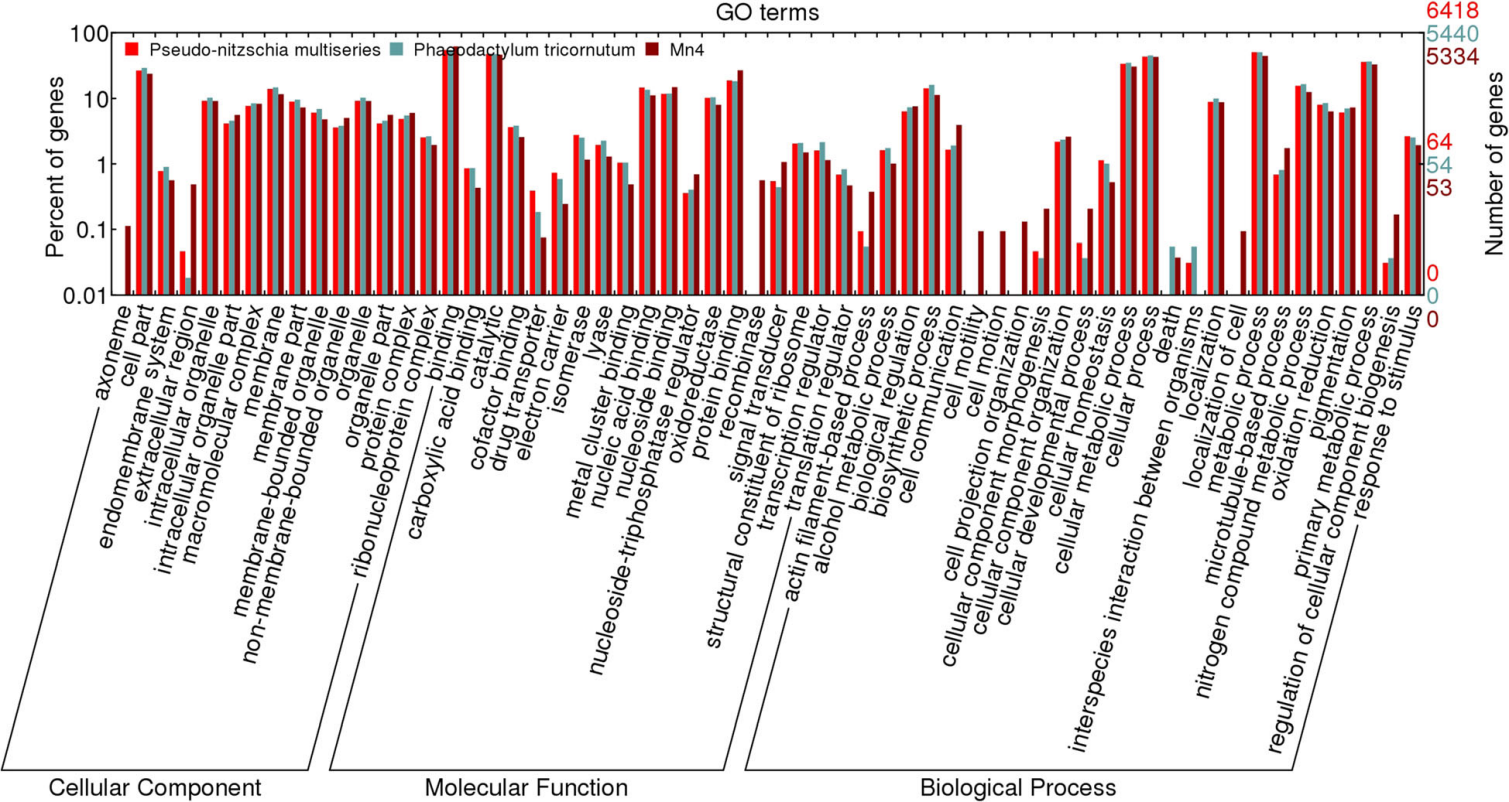
Statistics for the comparison of GO annotation of Mn4, *Pseudo-nitzschia multiseries* CLN-47 and *Phaeodactylum tricornutum*. All gene families are annotated into three parts: cellular component, molecular function, and biological process

In contrast to the two marine protists *Pseudo-nitzschia multiseries* CLN-47 and *Phaeodactylum tricormutum*, the endomembrane system of Mn4 was predicted to be relatively limited, and some metabolic activities associated with membrane structure were predicted to be reduced as well. There were significant fewer GO cellular component terms involved in membrane (GO:0012505, GO:0016020, GO:0044425) in the Mn4 genome annotation than those in the genomes of the two marine protists, including membrane-bounded organelle (GO:0043227) (Fig. 2 & Additional file 1: Table S3). However, the terms involved in organelle part (GO:0043226, GO:0044446) of Mn4 were enriched markedly, especially the non-membrane-bounded organelle (GO:0043228). Some metabolic activities independent of membrane structure tended to be more active in Mn4 than these protists, while others dependent on membrane decreased, such as oxidoreductase (GO:0016491), lyase (GO:0016829) and isomerase activities (GO:0016853).

The molecular mechanisms for motility of thraustochytrids may be different from *P. multiseries* CLN-47 and *P. tricornutum*. The strain Mn4 has fewer genes annotated with a response to chemical stimulus term (GO:0042221), however, signal transducer activity (GO:0004871) and cell communication (GO:0007154) were enriched and may mediate interactions with such as signaling, attaching to organisms, extracellular matrix, and other environmental variables through signaling or attaching. In addition, the relatively higher annotated content of cell motion (GO:0006928) and cell motility (GO:0048870) suggests that the strain Mn4 may have a capacity to control self-propelled movements, which could enable the translocation of its cells in marine environments. This is consistent with these unicellular organisms’ abilities to live independently in marine environments.

### Phylogenic analysis

**Fig. 3:**
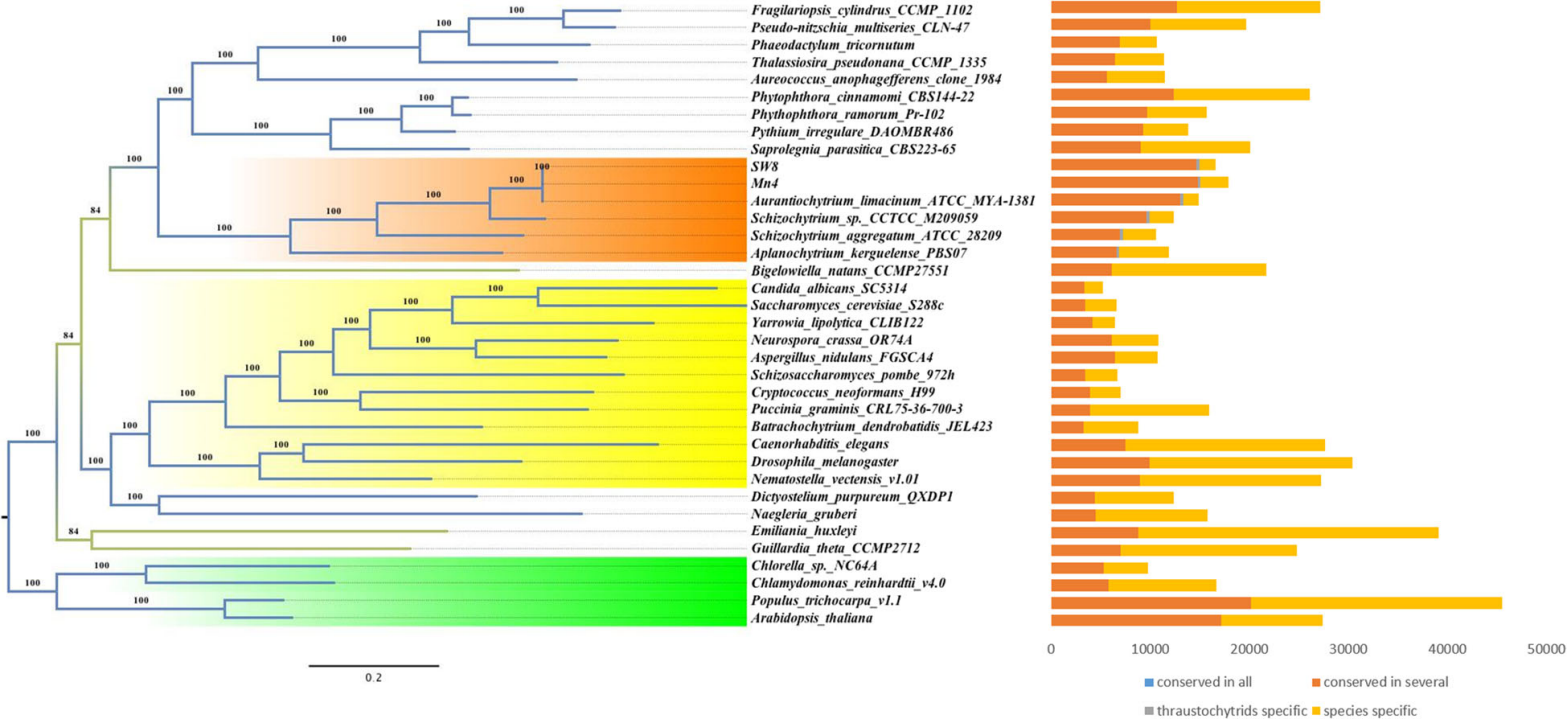
Phylogenetic relationship and gene conservation of the thraustochytrids and other species

Thirty-six eukaryotic species (Additional file 1: Table S4), including SW8 and Mn4, members of the Stramenopiles (phylogenetically related to thraustochytrids), fungi, plants and animals were selected to assess the phylogenic placement of these lineages. A phylogenetic tree was constructed from 1024 orthologous gene groups that contained genomes of at least 20 species (Fig. 3). Only 9 single-copy orthologous gene groups were found in the genomes of 36 species. There were 273 groups completely unique to the thraustochytrids. The strains Mn4, SW8, and MYA-1381 clustered together on the phylogenetic tree. Thus, our results support hypothesis that speciation occurred earlier for *A. kerguelense* PBS07, which has the earlier speciation than that of *S. aggregatum* ATCC 28209, with latest speciation for DHA-producing strains.

To better understand the evolution of thraustochytrids, an evaluation for whole-genome duplication (WGD)/segmental duplicates was applied with the MCScanX algorithm (Additional file 1: Table S5). The percentage of WGD duplicates in the DHA-producing thraustochytrids genomes ranged from 78 to 88%, but the ratio of non DHA-producing strains was only 38 and 6% for *S. aggregatum* ATCC 28209 and *A. kerguelense* PBS07, respectively. Thus, *S. aggregatum* ATCC 28209 may have experienced at least one more time WGD duplicate event than *A. kerguelense* PBS07, and so do the DHA-producing strains compared to *S. aggregatum* ATCC 28209. Therefore, from evolutionary point of view, the function for DHA production was obtained in the later evolutionary processes.

### Gene family analysis

**Fig. 4:**
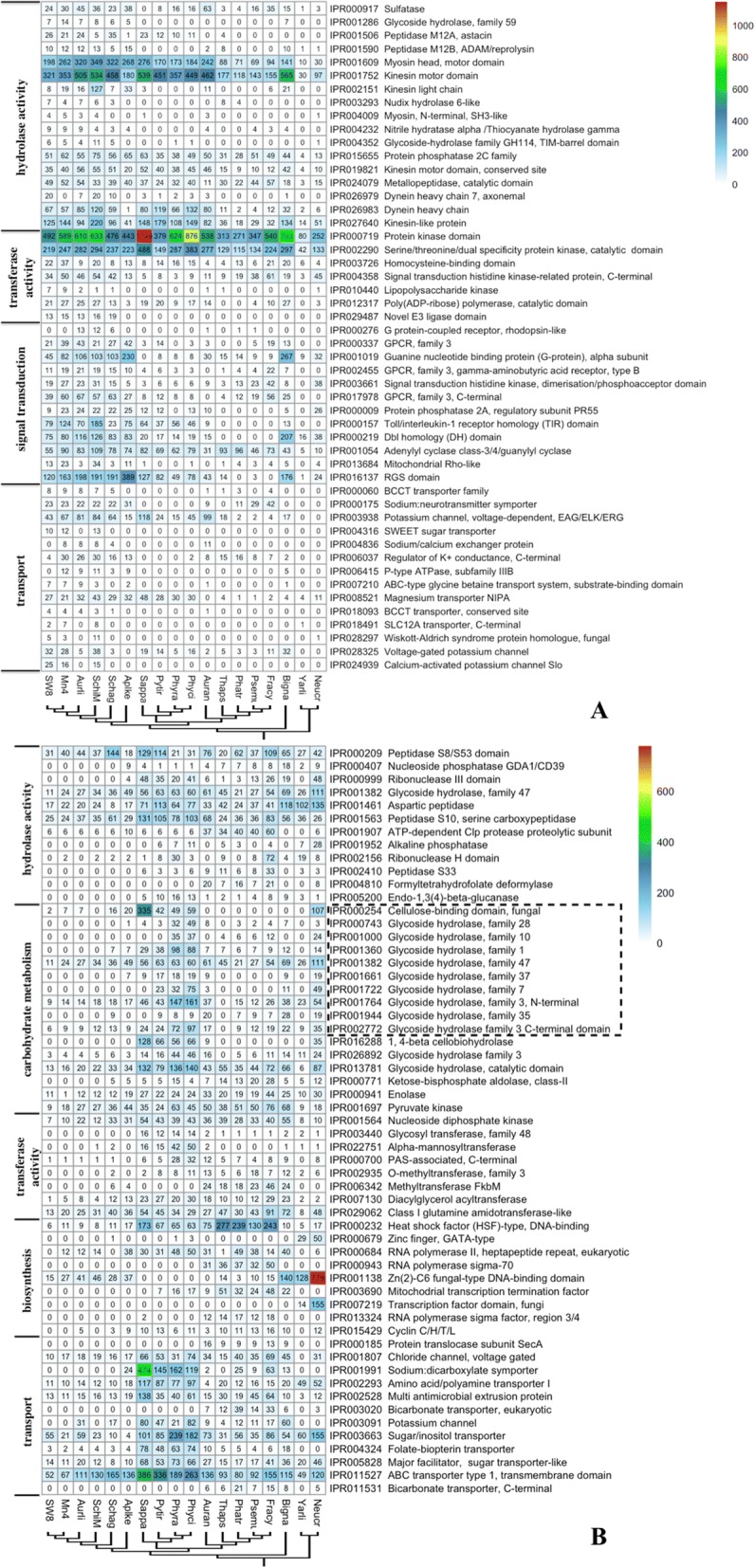
(**a**) IPR domains enriched in thraustochytrids compared to outgroups; (**b**) IPR domains depleted in thraustochytrids compared to outgroups. Values are colored along a white (low) to red (high) color scale, with color scaling relative to the low and high values of each row

To understand the (gain or loss) dynamics of functional domains in the genomes of thraustochytrids, comparison of InterPro categories was carried out among thraustochytrids and 12 outgroups, including 2 fungi. Two of the selected outgroup species, *Phaeodactylum tricornutum* and *Yarrowia lipolytica* CLIB122, were reported to produce PUFAs [17, 18]. Out of 9093 total IPR domains examined with at least one copy in any proteome set, 51 significantly (FDR *P*-value ≤0.05) enriched IPR domains were classified primarily into four GO functional categories: hydrolase activity, transferase activity, transport, and signal transduction (Fig. 4A & Additional file 1: Table S6). Furthermore, signal transduction, especially guanine nucleotide binding proteins (G proteins) and G protein-coupled receptors (GPCRs) were significantly enriched in thraustochytrids. There is one G protein domain (IPR001019) and three GPCR domains (IPR000337, IPR002455 and IPR017978) highly enriched (FDR *P*-value ≤0.05) in thraustochytrids’ genomes. Genes containing these domains were selected for further gene tree phylogenetic analysis (Additional file 3: Figure S2, Additional file 4: Figure S3, Additional file 5: Figure S4 and Additional file 6: Figure S5). The IPR001019 domain, gene duplications were formed before speciation of various thraustochytrids. However, the phylogenetic trees suggest that genes related to IPR000337, IPR002455 and IPR017978 domains were duplicated after speciation of DHA-producing thraustochytrids. In another word, G protein and GPCRs diversity may attribute to the speciation event and the gaining ability of DHA production for thraustochytrids, respectively.

Compared to the outgroup species, 60 IPR domains were significantly (FDR P-value ≤0.05) depleted in the genomes of thraustochytrids and assigned into 6 groups: hydrolase activity, transferase activity, transport, arbohydrate metabolism and biosynthesis (Fig. 4B & Additional file 1: Table S7). There were 10 domains in carbohydrate metabolism with hydrolase activity (circled by a dash line on Fig. 4B). Sixteen CAZymes were noticeably absent in the genomes of thraustochytrids and primarily belonged to the glycoside hydrolase (GH) family, which participates in decomposition of the major components of plant cell walls, including cellulose, hemicellulose, pectin or trehalose. Another absent set of IPR domains encoded several types of sugar transporters, such as IPR003663 (sugar/inositol transporter) and IPR005828 (major sugar transporter-like facilitator). These absences suggest their adaptation to the infrequent use of saccharides from marine plant material and/or other sugar substrates (e.g., carbohydrates, organic alcohols, and acids). Cellulose-binding domains (IPR000254) is typically a hallmark of fungi that utilize plant materials for nutrients [19]. At this point, there is no solid evidence to support plant cell wall degradation function of thraustochytrids in vivo. Our findings support the hypothesis of thraustochytrid’s nutrient model as ‘left-over’ scavengers [20].

### Secreted CAZy enzymes

Thraustochytrids have been reported to have the capacity to break down organic matter in marine, including extracellular carbohydrates. It contradicts with our result of the reduced copies of IPR carbohydrate metabolism genes in their genomes. To further investigate details of carbohydrate metabolism in the secretomes of thraustochytrids, we explored the predicted secretomes using the curated CAZymes domains. The number of predicted secreted proteins in the genomes of thraustochytrids ranged from 252 to 330 with no significant difference among those of DHA-producing or non DHA-producing strains (Table 2). Seventy secretome gene families were found in more than 4 genomes and thus were likely present in the ancestor genomes or part of the ancestral secretome of thraustochytrids. However, only 2 secreted gene families were present in all 6 thraustochytrid genomes. Of 70 secretome gene families, more (ranging from 54 to 68) were detected in the genomes of DHA-producing strains than those of non DHA-producing ones (only 24–33). Therefore, the sibship among DHA-producing strains was closer than that of non DHA-producing strains. Overall, results of predicted secretome analysis was consistent with the results of phylogenetic tree (Fig. 3).

**Table 2:** Distribution of CAZy Families in secretomes/genomes of thraustochytrids

Species		Mn4	SW8	Aurli	SchiM	Schag	Aplke
Total secretome		297	252	324	330	297	316
Number of orthologous groups		219	190	239	160	86	66
Number of ancestral groups		61	54	68	61	33	24
Number of no orthologous groups		78	62	85	170	211	250
CAZymes in secretomes/genomes	CE	1/62	1/56	1/52	3/56	3/48	1/67
	GH	11/53	12/57	3/49	13/55	8/60	17/61
	GT	8/147	10/143	8/122	8/140	12/97	11/151
	PL	0/0	0/0	0/0	0/1	0/1	0/0
	CBM	1/24	0/22	1/17	1/14	0/20	3/17
	AA	0/25	0/28	0/23	1/21	0/24	2/22

The CAZymes of thraustochytrid genomes and secretomes were classified into five CAZy classes (Table 2, Additional file 1: Tables S8 & S9): glycoside hydrolases (GH), glycosyltransferases (GT), polysaccharide lyses (PL), carbohydrate esterases (CE) and carbohydrate binding modules (CBM). No gene for polysaccharide lyases (PL) was found in the secretomes. At least, some of thraustochytrids is unlikely to degrade polysaccharides of plant cell wall and other resources in marine. This seems to concur with our results that both the strains Mn4 and SW8 did not grow well in the media containing only either starch or carboxymethycellulose sodium (CMC-Na) as the sole carbon source (Wang et al., unpublished data). Most CAZymes of the predicted secretomes were the members of GH and GT families. Six of GH (GH3, GH30, GH43, GH59, GH92 and GH114) family and GT (GT1, GT23, GT25, GT32, GT41 and GT68) were found in predicted secretomes of more than 3 thraustochytrid genomes (Additional file 1: Table S9). Of these enzymes, GH3 participates in the degradation of lignin and xylan and GH43 in the breakdown of pectin and xylan. Overall, the number of secretomes for these CAZymes was low due to the obvious deletion of their functional blocks in the genomes of thraustochytrids.

**Fig. 5:**
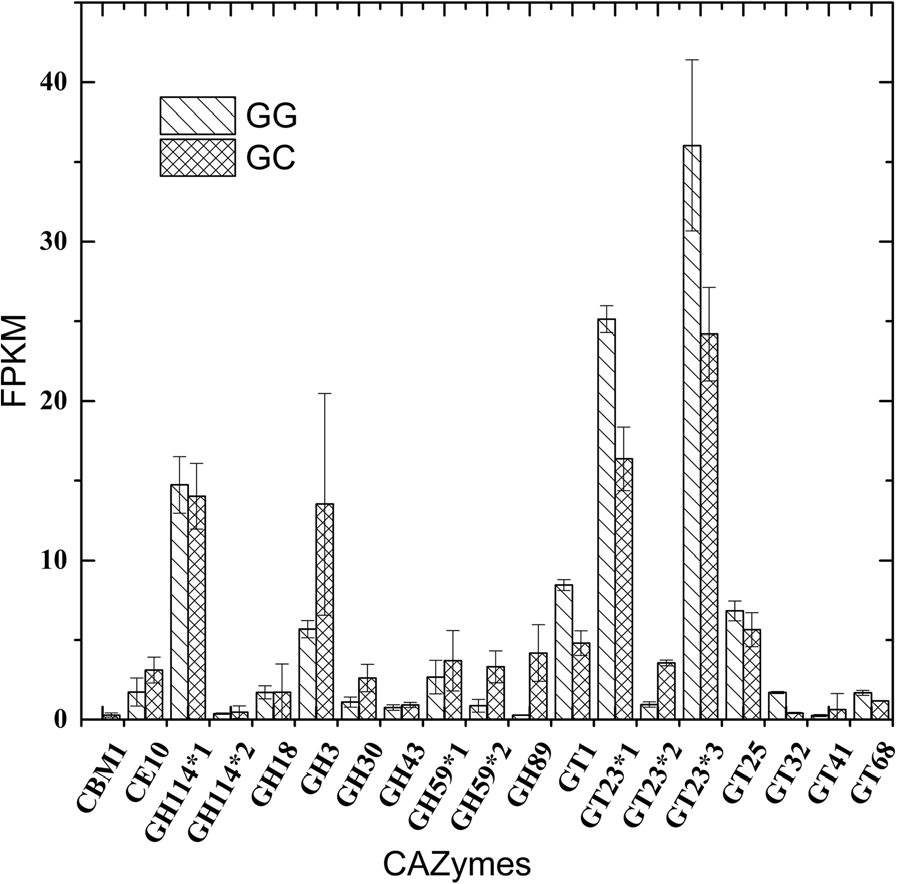
Comparison of expression levels for CAZymes of Mn4 cultivated with glucose and cellulose, as the carbon source. Genes in the same CAZymes family are numbered with an asterisk

After comparing the transcriptional levels of the strain Mn4 grown in two kinds of culture media (GG-vs-GC), a total of 204 unigenes with obvious different expression levels were identified by sequence alignment (Additional file 1: Table S10). Of these genes, no expression difference for CAZymes genes were detected for the cells grown on glucose and cellulose. However, except GH92, 11 of GH and GT families (Additional file 1: Table S9) and the family GH89 were detected to be expressed at low, but varied levels (Fig. 5 & Additional file 1: Table S11). The transcriptome data further indicated that the strain Mn4 may not have a capability of degrading organic matters.

### DHA synthesis pathway

The pathway of DHA biosynthesis in thraustochytrids has not been completely unraveled. Two biosynthetic pathways, i.e. the conventional fatty acid synthesis (FAS) route and the polyketide synthase (PKS) system, have long been speculated to exist in the genomes of thraustochytrids [21].

**Fig. 6:**
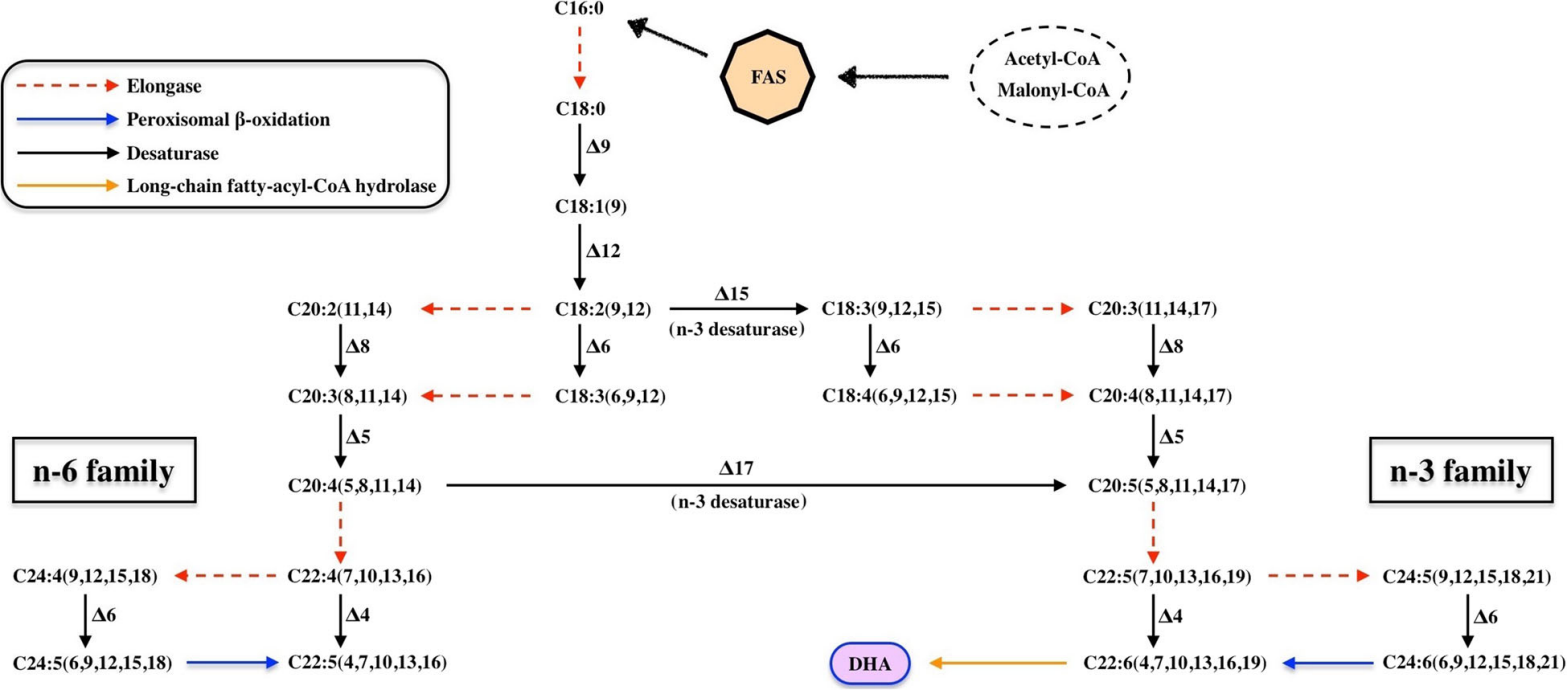
The predicted FAS pathway for the DHA biosynthesis in KEGG. The stearic acid (C16:0) is successively desaturated and elongated through a series of reactions leading to the formation of n-3 and n-6 PUFAs. There exist n-3 desaturases that can convert n-6 PUFAs into DHA

The longest products of FAS pathway commonly found in almost all organisms are either C16 or C18 long-chain saturated fatty acids [22]. In thraustochytrids, these fatty acids are then modified through a sequencial processes of enzyme catalysis to extend the carbon chain for long-chain DHA (C22:6) production (Fig. 6). Enzymes related to the FAS route were primarily from the map01040 pathway in KEGG, which contained elongase, desaturase, peroxisomal β-oxidation and long-chain fatty-acyl-CoA hydrolase. Some enzymes related to DHA synthesis were added in to complete the FAS route. Elongase and peroxisomal β-oxidation contain four steps as one functional module. The long-chain fatty-acyl-CoA hydrolase (EC 3.1.2.2) hydrolyzed CoA thioesters of DHA and other long-chain fatty acids to achieve their final products. The FAS route included no less than seven types of desaturase, delta-4, delta-5, delta-6, delta-8, delta-9, delta-12 and n-3 (e.g. delta-15 and delta 17). Our analyses of the map01040 pathway in KEGG, the whole genome annotation of 6 thraustochytrid strain and two reference strains (*P. tricornutum* (protist) and *Y. lipolytica* (fungi)) suggest that sufficient enzymes of elongase, peroxisomal β-oxidation and long-chain fatty-acyl-CoA hydrolase were present in the genome of the strains Mn4 (Table 3). Both delta-6 and delta-8 desaturases were found to be abundant in the genomes of all thraustochytrid strains and the references. Delta-4 desaturase was also detected in the genomes of all analyzed genomes except in that of *Y. lipolytica* CLIB122. Relatively low copies of (< 3 copies in each individual genome) delta-9 and delta-12 desaturases were detected in all the genomes. Moreover, delta-5 desaturase gene was present only in 5 genomes of thraustochytrid strains and absent in the genomes of *A. limacinum* ATCC MYA-1381 and the two references. In other words, only half of DHA-producing strains (four thraustochytrids and the references) had delta-5 desaturases. Particularly, no gene of n-3 desaturases, which is the essential enzyme for DHA production, were detected in the genomes of DHA-producing thraustochytrids strains.

**Table 3:** Enzymes involved in the DHA biosynthesis of the FAS pathway

Enzymes		EC No.	KO	Mn4	SW8	Aurli	SchiM	Schag	Aplke	Phatr	Yarli
elongase	E1	2.3.1.199	K10203	2	2	2	2	3	1	2	0
			K10246	1	1	1	1	0	0	0	1
	E2	1.1.1.330	K10251	3	3	3	5	2	6	9	1
		1.1.1.100	K00059	87	87	89	75	78	102	63	47
	E3	4.2.1.134	K10703	1	1	1	2	1	1	1	1
			K18880	2	2	2	2	2	7	0	5
	E4	1.3.1.93	K10258	4	3	3	2	4	8	3	2
		1.3.1.38	K07753	2	2	2	3	0	3	2	3
desaturase	△9	1.14.19.1	K00507	1	1	1	1	1	1	1	1
		1.14.19.2 1.14.19.11 1.14.19.26	K03921	0	0	0	0	0	0	1	0
		1.14.19.2	K03922	0	0	0	0	0	0	0	0
	△12	1.14.19.23 1.14.19.13	K10255	2	2	2	1	2	2	2	1
		1.14.19.6 1.14.19.22	K10256	0	0	0	1	1	0	2	1
	n-3	1.14.19.25 1.14.19.35 1.14.19.36	K10257	0	0	0	0	0	1	1	0
		1.14.19.13	K21707	0	0	0	0	0	0	0	0
	△6	1.14.19.2	K00508	0	0	0	0	0	0	0	0
		1.14.19.3	K10226	17	14	16	13	16	18	5	3
	△8	1.14.19.4	K13076	8	9	9	9	13	6	4	4
			K21732	0	0	0	0	0	0	0	0
	△5	1.14.19.44	K10224	1	1	0	2	1	3	0	0
	△4	1.14.19.31	K12418	9	2	2	5	8	11	4	0
peroxisomal β-oxidation	P1	1.3.3.6	K00232	19	20	19	19	16	24	7	10
	P2	4.2.1.17	K01825	1	1	0	0	0	0	1	0
			K01782	15	17	16	16	12	21	6	3
			K07515	1	1	1	1	1	1	1	0
	P3	1.1.1.211									
			K10527	11	13	11	11	12	15	5	3
	P4	2.3.1.16	K07513	11	12	11	12	8	14	6	4
long-chain fatty-acyl-CoA hydrolase		3.1.2.2	K01068	8	6	5	7	4	2	0	1

In order to investigate the expression level of key enzymes involved in the DHA biosynthesis in thraustochytrids, the transcriptome data of the strain Mn4 were further used to decipher the DHA biosynthesis pathway. No significant difference at the expression level for FAS system was detected for cell grown at medium containing glucose and cellulose as carbon sources (Additional file 1: Table S12). The transcriptome data indicate that the expression level of delta-6, delta-8 and delta-12 desaturases were significantly high (FPKM > 100). The gene of delta-9 desaturase was also detected to be high with the FPKM of 83.73. Thus, these four desaturases are likely involved in the DHA biosynthesis of the strain Mn4. On the other hand, the genes of delta-4 and delta-5 desaturases, were expressed at a low level (FPKM < 5). Particularly, no expression of n-3 desaturase gene was detected. Overall, the absence of n-3 desaturase and the low expression level of delta-4 and delta-5 suggest that DHA biosynthesis unlikely occurs through the FAS route in the strain Mn4.

It has been proposed that DHA can be biosynthesized through the PKS pathway using 8 domains: 3-ketoacyl synthase (KS), malonyl-CoA:ACP acyltransferase (MAT), acyl carrier protein (ACP), 3-ketoacyl-ACP reductase (KR), acyltransferase (AT), chain length factor (CLF), enoyl reducatase (ER) and a dehydrase/isomerase (DH) [21, 22]. The PKS biosynthetic pathway and other secondary metabolism-specific gene clusters (e.g. nonribosomal peptide synthetase, terpene cluster and arylpolyene clusters) were detected using the antiSMASH [23]. Interestingly, 9 PKS-like gene clusters were detected in the genomes of DHA-producing strains Mn4, SW8, and CCTCC M209059 and 10 PKS-like gene clusters observed in the genome of ATCC MYA-1381 (Additional file 1: Table S13). However, only 1 PKS-like gene cluster was detected in the genomes of non DHA-producing strains PBS07 and ATCC 28209. If the PKS-dependent DHA biosynthesis occurs in thraustochytrids, PKS-like gene clusters should be collinear with those of the other DHA-producing strains on their own chromosomes and have similar functional annotation for their core and accessory genes. Only three PKS-like gene clusters with the same functional annotation were detected in the syntenic regions of DHA-producing strains. Nevertheless, several essential components (e.g. ER and DH) were absent from these 3 gene clusters, which did not match with the predicted PKS pathway [22].

Furthermore, 16 PKS-like genes were detected in the genome of strain Mn4 using InterPro domain annotations (Table 4). Of these genes, 3 contained a complete PKS backbone, 2 without AT domain but with more than one KS domain, 1 with only KS domain, and 10 only with trans-AT domain. The complete PKS backbone gene Mn4_10926 with AT-KS-PP domain arrangement, which lacked the essential modification domains (e.g. DH, KR, and ER), had a high expression level (FPKM > 100). Furthermore, because the genes Mn4_10535 (complete) and Mn4_04057 (without AT domain) contained DH and KR domains, they likely had the capability to synthesize more complex compounds through adding two-carbon unit. However, transcriptional analysis revealed their low expression (FPKM < 20 for Mn4_10535 and FPKM < 10 for Mn4_04057). Finally, of all the PKS-like genes, Mn4_04057 (PP-KS-DH-KR-PP:KS-KR-PP-KS-DH-KR-PP:KS-MT-KR-PP-TE) contained the most functional domains, it still did not contain the all required for DHA biosynthesis. Thus, no inclusive evidence was found to support that the complete PKS-dependent DHA biosynthetic pathways were present in the genomes of Mn4.

**Table 4:** Polyketide synthase backbone genes in Mn4

Gene ID	Secondary Metabolite type	Domain arrangement^a^	Counts	FPKM
Mn4_07902	PKS	AT-AT-AT-KS-PP	1411.17	15.05
Mn4_10535	PKS	KS-AT-PP-PP-PP-PP-PP-PP-PP-KR-DH	613.52	10.10
Mn4_10926	PKS	AT-KS-PP	13,515.00	308.75
Mn4_04057	PKS-like	PP-KS-DH-KR-PP:KS-KR-PP-KS-DH-KR-PP:KS-MT-KR-PP-TE	465.00	3.52
Mn4_13634	PKS-like	KS-KS-AT-AT	348.67	8.01
Mn4_03582	KS-only	KS	169.00	17.70
Mn4_00666	trans-AT	AT-AT	1568.00	100.42
Mn4_01567	trans-AT	AT-AT	446.67	21.99
Mn4_01870	trans-AT	AT	265.00	25.17
Mn4_01972	trans-AT	AT	63.34	7.31
Mn4_04434	trans-AT	AT-AT	576.33	17.42
Mn4_07257	trans-AT	AT-AT	536.17	43.96
Mn4_08818	trans-AT	AT	9.33	0.97
Mn4_10925	trans-AT	AT	37.34	5.51
Mn4_11592	trans-AT	AT	1699.17	79.82
Mn4_14122	trans-AT	AT	70.34	3.63

^a^The colon means there is an overlapping between two domains. (*AT* Acyl transferase, *DH* Dehydratase, *KR* Keto reductase, *KS* Beta-ketoacyl synthase, *MT* Methyltransferase, *PP* Phosphopantetheine, *TE* Thioesterase)

## Discussion

Highly accurate genomes of two strains (SW8 and Mn4) assembled from second generation short read Illumina and single-molecule PacBio sequencing has provided unprecedented genetic evidence on carbon utilization strategy and the molecular pathway of DHA biosynthesis for this interesting group of fungal-like marine protists.

The genomic analysis of CAZy secretory proteins content yielded unexpected results. Thraustochytrids have been reported to produce a battery of extracellular hydrolytic enzymes, in vitro, such as amylase, cellulase, lipase, protease, phosphatase, pectinase, and xylanase [24–28] which are necessary for their ability to utilize highly refractory organic compounds of higher plants. CAZy classes of functional domains are depleted in thraustochytrids’secretomes and not enough for degrading carbohydrates in the environment (Table 2 & Additional file 1: Table S9). Furthermore, there are 19 secreted CAZymes detected in the strain Mn4. The expression level of these enzymes in the transcriptome of Mn4 were very low (Additional file 1: Table S11). Our findings appear to contradict with the common view on the thraustochytrid’s ability of utilizing detrital plant materials or highly refractory organic matters as nutrient resources. However, it seems to be consistent with the report that not all thraustochytrids produce cellulases [4]. It is also consistent with our recent findings that Mn4, SW8 and several other lab thraustochytrid strains did not grow well in the media containing only either starch or CMC-Na as the sole carbon source (Wang et al., unpublished data). In addition, some members of thraustochytrids were unable to produce chitinase and thus may not degrade zooplankton exoskeletons [29]. Furthermore, thraustochytrids generally co-occupy spatial niches with bacteria. They do not have reported antagonostic interactions with bacteria or produce antibacterial substances, neither appear to produce special enzymes not found in bacteria [30]. Several experimental observations suggest that thraustochytrids compete with bacteria for nutrients and grew faster in the absence of bacteria, but also have robust growth on bacterially-colonized substrates [20]. One hypothesis about their interactions suggests that the thraustochytrids feed upon residual nutrients left over by bacteria. Our results support idea that some of thraustochytrids may subsist on ‘left-over scavenging’ of nutrient substrates following bacterial growth. There may exist two types of saprobic thraustochytrids in marine environments. One group is incapable of producing the complete set of cellulases and employs the “left-over scavenging” model to live in marine environments. The other group, capable of producing a more complete repertoire of cellulases, can utilize plant or other refractory organic matters for a nutrient source. Detailed genomic information on the second group of thraustochytrids would shine more light on the living style of thraustochytrids. Clearly, the living-style or nutrient model of thraustochytrids in marine environments remains one of the most fascinating and interesting microbial topics in marine ecosystems.

Some thraustochytrid strains produce omega-3 PUFAs, i.e., DHA and EPA [30]. Biochemical studies to characterize individual enzymes from the standard FAS and PKS pathways have been employed to better understanding their biosynthetic mechanisms [31]. Currently there is no evidence to support the hypothesis that biosynthesis of DHA is via either of two conjectural pathways. The two major steps in fatty acids biosynthesis are elongation and desaturation carried out by elongases and desaturases, respectively, through FAS pathway. The longest end products of FAS pathway are either C16:0 or C18:0 saturated fatty acids through the FAS pathway (map01212, http://www.genome.jp/kegg-bin/show_pathway?ko01212). These two fatty acids are then modified through desaturations and elongations for the production of an extended range of unsaturated fatty acids or PUFAs [22]. The desaturases delta-15 (n-3), delta-5 and delta-4 catalyze the formation of C20:4, C22:5, and C22:6, respectively. The desaturase delta-5 is essential for the biosynthesis of EPA and the precursor of DHA and the delta 4 for that of DHA in the pathway. Based on RNASeq and analysis of the transcriptome of Mn4, expression of the n-3 desaturase (delta-15 and delta-17) was not detected and the latter two desaturases (delta-4 and delta-5) were expressed at very low level. However, the remaining of desaturases displayed very high expression level. The n-3 desaturase is absent in the genomes of DHA-producing strains of thraustochytrids. Although the long-chain polyunsaturated fatty acids, omega-6 PUFAs, can be synthesized in the absence of the n-3 desaturase, but none of omega-3 PUFAs (e.g. DHA) can be formed through FAS pathway. The results of this study i.e., genomic and transcriptomic analyses, did not support the involvement of the 3 essential desaturases (delta-4, delta-5 and n-3) in DHA biosynthesis through FAS pathway in the thraustochytrid strain (Table 3). Thus, the genomes of DHA-producing thraustochytrids may not contain the complete FAS genes.

PUFAs are synthesized by acyl carrier protein (ACP) in the PKS pathway. ACP is used as a covalent attachment point for chain extension through reiterative cycles. During the full long-chain unsaturated fatty acid synthesis process, a series of enzymes including 3-ketoacyl synthase (KS), 3-ketoacyl-ACP reductase (KR), enoyl reducatase (ER), and dehydrase/isomerase (DH) are involved in the PKS pathway. Our search for gene clusters in the genomes of DHA-producing thraustochytrids did not reveal any complete cluster of PKS for PUFAs biosynthesis consistent with previous work [32]. Instead, several lines of evidence suggest the existence of an alternative mechanism, which involves both the FAS and PKS pathways, for DHA biosynthesis. Over 9 clusters of PKS-related genes containing KS domains were detected in the genomes of DHA-producing strains (Additional file 1: Table S13). The multiple copies of PKS-like clusters in these DHA-positive strains suggests that these genes may be important for biosynthesis of PUFAs. However, the clusters are fragmented indicating that DHA may not be synthesized through the classical PKS system alone. Although the complete PKS anchor gene (Mn4_10926) is expressed at a high level (FPKM > 100), it is not sufficient for PUFAs-synthesis without KR, ER and DH domains. In another word, multiple copies of PKS-like clusters and high-level expression of the PKS anchor gene suggest the involvement of an active PKS system in the biosynthesis of PUFAs. At the same time, lack of essential PKS or modification domains for those PKS-like genes suggest that PUFAs may be synthesized through a combination of different pathways in thraustochytrids.

Several key enzymes in FAS are similar to some functional domains of PKS pathway. For example, KS and KR are found in both FAS and PKS pathways. KS catalyzes the condensation of a wide range of substrates with varied carbon-length of saturated and unsaturated fatty acids, KR carries out the reductive modification of the growing polyketide and fatty acyl chains in FAS and PKS pathways, respectively. Thus, we hypothesize that a third pathway, which involves both FAS and PKS pathway, is involved for PUFAs biosynthesis in thraustochytrids. However, the mechanism underlying the catalytic functions of individual biochemical reactions remains to be defined.

## Conclusions

Phylogenomic analyses of high-quality thraustochytrid genomes have revealed that PUFAs-produced thraustochytrids are closely related to non-DHA producing strains. Comparative genomics of thraustochytrids and sister species shows that thraustochytrids have a reduced capacity for cellulose/hemicellulose degradation, but possesses expanded gene inventories for signal transduction and self-propelled movement. The distribution and diversity of secreted CAZymes suggests these some thraustochytrid strains do not produce substantial extracellular degrading enzymes. The identified FAS or the PKS pathways in these genomes were incomplete suggesting an unknown but novel process may exist in thraustochytrids for DHA production. Genomic and biochemical data support classification of thraustochytrids into two groups (i.e. DHA-producing and detritus-using) with different ecological functions. DHA-producing species likely have direct trophic interaction with other higher level trophic plankton in the food-web while the other primarily plays a role in nutrient cycling. As two groups may occupy different ecological niches in marine ecosystems, the genomic, biochemical, and phylogenomic comparisons allow for hypothesis development into the molecular and functional processes that contribute to their ecological roles.

## Methods

### Thraustochytrids cultures

Two strains *Schizochytrium* sp. PKU#Mn4 (JX847360) and *Thraustochytriidae* sp. PKU#SW8 (JX847378) were selected for genome sequencing. They were isolated from coastal marine habitats of Pearl River Delta region of China using the direct plating method [29] for mangrove leaf samples (Mn4) and the pine pollen-baiting method [33] from seawater samples (SW8). These stains were cultivated at 28 °C with reciprocal shaking (150 rpm). The culture medium contained glucose (20 g/L), peptone (1.5 g/L), yeast extract (1 g/L), and artificial seawater (NaCl 25 g/L, KCl 1 g/L, KH_2_PO_4_ 0.3 g/L, MgSO_4_·7H_2_O 5 g/L, NaHCO_3_ 0.1 g/L and CaCl_2_ 0.3 g/L). Both strains were shown to produce high yield of PUFAs using the GC method described previously and identified by identified by amplification and sequence analysis of complete 18S rRNA gene sequence [34].

### Genome sequencing and assembly

Genomic DNA was isolated from 100 ml of fresh culture. The cell suspension of Mn4 and SW8 strains was centrifuged at 13,200 rpm for 10 min. The resulting pellets were ground in liquid N_2_ to fine powder and washed once in 4.0 ml DNA extraction buffer (200 mM Tris-HCl, pH = 8.5; 250 mM NaCl; 25 mM EDTA, pH = 8.0; 0.5% SDS). Then, the pellets were suspended in 4.0 ml phenol:chloroform:isoamyl alcohol(25:24:1) and then centrifuged at 10,000 rpm for 15 min for twice. The supernatant was washed in equal volume chloroform and centrifuged at 13,200 rpm for 15 min. Genomic DNA was precipitated by adding 2.5 volume 100% ethanol and collected by spinning at 13,200 rpm, 4 °C for 10 min. After the supernatant was discarded, the resulting genomic DNA pellet was stored in 5.0 ml 70% cold ethanol at 4 °C for overnight to allow the impurity to dissolve. Finally, after discarding the supernatant, the resulting DNA pellet was air-dried for 10 min and dissolved in 0.4 ml autoclaved ddH_2_O with 100 μg/ml RNase*.*

Whole genome sequencing of Mn4 and SW8 strains was performed with the Illumina HiSeq 2500 HT System and PacBio RS System using SMRT Sequencing technology. Overall sequencing depth was calculated to be 60X coverage (40X Illumina and 20X PacBio) for each strain. There were 28,340,999 reads for Mn4 and 33,183,554 for the SW8. After PacBio reads were corrected by alignment of Illumina reads, the genomes were assembled with the PacBioToCA module of Celera Assembler v8.2 [35–37] with the “maxGap 50” setting to perform a hybrid assembly of the HiSeq data and PacBio data. Summary statistics of the assemblies are presented in Table 1. Assessment of genome completeness was performed with BUSCO using Eukaryotic models [15].

### Gene prediction and function analysis

Protein-coding genes were predicted using MAKER [38] that leveraged the programs SNAP, Augustus, and GeneMark-ES. These ab initio results were assessed and built into final gene annotations which scored and ranked ab initio gene model for consistency with protein homology to thraustochytrids proteins in the UniProt protein database [39]. CEGMA [40] was used for bootstrapping the training set for the ab initio tools. The MAKER predicted proteins were functionally annotated by InterProScan [41] analysis to assign GO terms, InterPro domains and signalP classifications.

The Mn4 strain genome annotation was used to compare thraustochytrids GO annotations with two other phylogenetically related marine protists *Pseudo-nitzschia multiseries* CLN-47 and *Phaeodactylum tricornutum* (Additional file 1: Table S3). A Pearson Chi-Square test was used to compare the numbers of each ontology types for the two newly sequenced thraustochytrid genomes. A significance level of *p*-value ≤0.05 was used to determine under- or over-represented GO terms.

Significant differences in domain content between genomes were found using the hypergeometric distribution to compare the number of InterPro (IPR) domains in thraustochytrids and other 12 genomes as implemented in the R package pheatmap. The results were filtered using a q value (set to 5% allowable FDR) to account for multiple testing [42]. Finally, these domains were mapped to GO annotations and classified primarily into different functional categories using Generic GO Slims (http://www.geneontology.org/GO.slims.shtml).

### Public data for comparative analyses

Genome assemblies and annotations of 34 other organisms (Additional file 1: Table S4) were used in this study. Of them, 17 (*Aplanochytrium kerguelense* PBS07, *Aurantiochytrium limacinum* ATCC MYA-1381, *Schizochytrium aggregatum* ATCC 28209, *Pseudo-nitzschia multiseries* CLN-47, *Phaeodactylum tricornutum*, *Fragilariopsis cylindrus* CCMP 1102, *Thalassiosira pseudonana* CCMP 1335, *Aureococcus anophagefferens* clone 1984, *Bigelowiella natans* CCMP 2755, *Emiliania huxleyi*, *Guillardia theta* CCMP2712, *Naegleria gruberi*, *Dictyostelium purpureum* QXDP1, *Nematostella vectensis*, *Chlamydomonas reinhardtii*, *Chlorella* sp. NC64A, *Populus trichocarpa*) are available in the JGI [43], 12 (*Phytophthora cinnamomi* CBS144–22, *Phythophthora ramorum* Pr-102, *Saprolegnia parasitica* CBS223–65, *Pythium irregulare* DAOMBR486, *Saccharomyces cerevisiae* S288c, *Schizosaccharomyces pombe* 972 h, *Candida albicans* SC5314, *Aspergillus nidulans* FGSCA4, *Neurospora crassa* OR74A, *Puccinia graminis* CRL75–36–700-3, *Batrachochytrium dendrobatidis* JEL423, *Cryptococcus neoformans* H99) in FungiDB (http://FungiDB.org/FungiDB) [44] and 5 (*Schizochytrium* sp. CCTCC M209059, *Yarrowia lipolytica* CLIB122, *Drosophila melanogaster*, *Caenorhabditis elegans*, *Arabidopsis thaliana*) in NCBI BioProject database (https://www.ncbi.nlm.nih.gov/bioproject) [45].

### Homology and phylogenetic analysis

Orthologous and paralogous gene families were identified with Orthologous MAtrix (OMA) [46] among 6 thraustochytrids and 30 additional complete genomes. To infer phylogenetic relationships, single copy gene families that contained more than 20 species were selected from the clustering and the proteins aligned using MUSCLE [47]. These alignments were concatenated and a maximal likelihood phylogenetic tree computed with RAxML [48] using the PROTGAMMAAUTO model of the amino acid substitution and 1000 bootstrap replicates. To better understand the evolution of thraustochytrids, the gene similarity and synteny analysis using the MCScanX algorithm classified all genes into 5 groups: singletons, dispersed duplicates, proximal duplicates, tandem duplicates, and whole-genome duplication WGD/segmental duplicates. WGD/segmental duplicates were inferred by the anchor genes in collinear blocks [49].

### Identification and annotation of predicted thraustochytrid secretome

To identify proteins belonging to the secretomes of all 6 thraustochytrids, the predicted proteins from the genome annotations were analyzed by three programs, TargetP v1.1 [50], WoLF PSORT [51], and TMHMM v2.0 [52] in addition to the secretion signals predicted by InterProScan. For a protein to be included in the putative secretome it must have a localization of “S” in TargetP, a WoLF PSORT annotation of extracellular, and no transmembrane regions after the signal peptide. These programs were run successively, in the order above, removing failed proteins at each step. TargetP was run using the “nonplant” setting and WoLF PSORT was run with the “fungi” setting as in [53]. CAZymes were annotated by submitting all protein-coding genes or secreted protein genes from each genome to the dbCAN webserver [54] available at http://csbl.bmb.uga.edu/dbCAN/annotate.php.

### Transcriptome analysis

Due to the high similarity of genomes feature and cell phenotype between the strains Mn4 and SW8, Mn4 was selected as the representative for transcriptome analysis. The strain Mn4 was cultivated at 28 °C with reciprocal shaking (150 rpm) using glucose as carbon source for 2 days which was the middle of the logarithmic phase of growth. Then, half of samples were transferred to another culture medium using cellulose as carbon source for another 3 days (GC), while the other samples were kept incubating in glucose culture medium (GG). Subsequently, total RNA was extracted using TRNzol Reagent Kit. RNA-Seq libraries were constructed using RNA Seq Library Preparation Kit and sequenced using Illumina HiSeq™ 2500. Reads were aligned to reference genome using TopHat2 [55]. The R package, edgeR [56], was used to identify differentially expressed genes (DEGs) between two treatments. EdgeR offers a rigorous statistical test for thresholded hypotheses under the GLM (Generalized linear model) framework. An absolute value of the log2 ratio of ≥2 and a false discovery rate (FDR) of ≤0.05 were used as the thresholds to judge the DEGs.

### Mechanisms of fatty acid synthesis

A set of the homologs of each enzyme in the FAS pathway were downloaded from pathway map01040 annotated as “biosynthesis of unsaturated fatty acids” (http://www.genome.jp/kegg-bin/show_pathway?map=map01040) in KEGG database to build a Hidden Markov Model (HMM) database using the “hmmbuild” and “hmmpress” command. Seven novel enzymes were added into the database based on their functional annotation information. The predicted proteomes were analyzed using the HMM database and the “hmmscan” command (e-values ≤1e-5). The genomes of *P. tricornutum* (protist) and *Y. lipolytica* (fungi) were selected as positive control reference species since they can both produce DHA [17, 19].

The AntiSMASH pipeline [23] with HMM signatures was used to identify and annotate putative polyketide synthase (PKS), nonribosomal peptide synthetase (NRPS), and terpene synthase (TPS) genes and gene clusters, and to predict the PKS and NRPS domain architecture in all 6 genomes. The gene order and conservation of clusters were manually inspected with the gene cluster alignment results from AntiSMASH website. Additionally, secondary metabolite anchor genes (called SM backbone genes) were predicted according to the annotations of InterPro domains.

## Additional files

Additional file 1: **Table S1.** Summary of genome sequencing quality and reads mapping quality. **Table S2.** Comparison of quality assessment and assembly statistics of two methods. **Table S3.** Comparison of GO classifications of annotated genes for *P. multiseries*, *P. tricornutum* and Mn4. **Table S4.** Genomes for the other organisms in this study. **Table S5.** Classification of duplicate gene origins in the thraustochytrids’ genomes. **Table S6.** Statistics for IPR domains enriched in thraustochytrids compared to outgroups. **Table S7.** Statistics for IPR domains depleted in thraustochytrids compared to outgroups. **Table S8.** Distribution of CAZy Families in genomes of thraustochytrids. **Table S9.** Distribution of CAZy Families in secretomes of thraustochytrids. **Table S10.** Statistics for the up-regulated /down-regulated genes identified in two different groups. **Table S11.** The transcriptional expression levels of secreted CAZyme genes in Mn4. **Table S12.** Expression of genes related to DHA biosynthesis of the FAS pathway in Mn4. **Table S13.** Summary of polyketide synthase (PKS) gene clusters in thraustochytrids. (XLSX 101 kb)

Additional file 2: **Figure S1.** Statistics for GO annotation of thraustochytrid strains Mn4 and SW8. (JPG 250 kb)

Additional file 3: **Figure S2.** Phylogenetic tree of all proteins containing IPR001019 domains. Total 18 species were assigned into 3 colored groups: pink for DHA-producing thraustochytrids, blue for non DHA-producing thraustochytrids and orange for 12 non thraustochytrids species. (JPG 217 kb)

Additional file 4: **Figure S3.** Phylogenetic tree of all proteins containing IPR000337 domains. (JPG 103 kb)

Additional file 5: **Figure S4.** Phylogenetic tree of all proteins containing IPR002455 domains. (JPG 215 kb)

Additional file 6: **Figure S5.** Phylogenetic tree of all proteins containing IPR017978 domains. (JPG 406 kb)
