# Supplementary figures and images for: Comparative analysis reveals unexpected genome features of newly isolated Thraustochytrids strains: on ecological function and PUFAs biosynthesis
Source: BMC Genomics. 2018 Jul 17;19:541. doi: 10.1186/s12864-018-4904-6 (PMC6050695; doi:10.1186/s12864-018-4904-6)

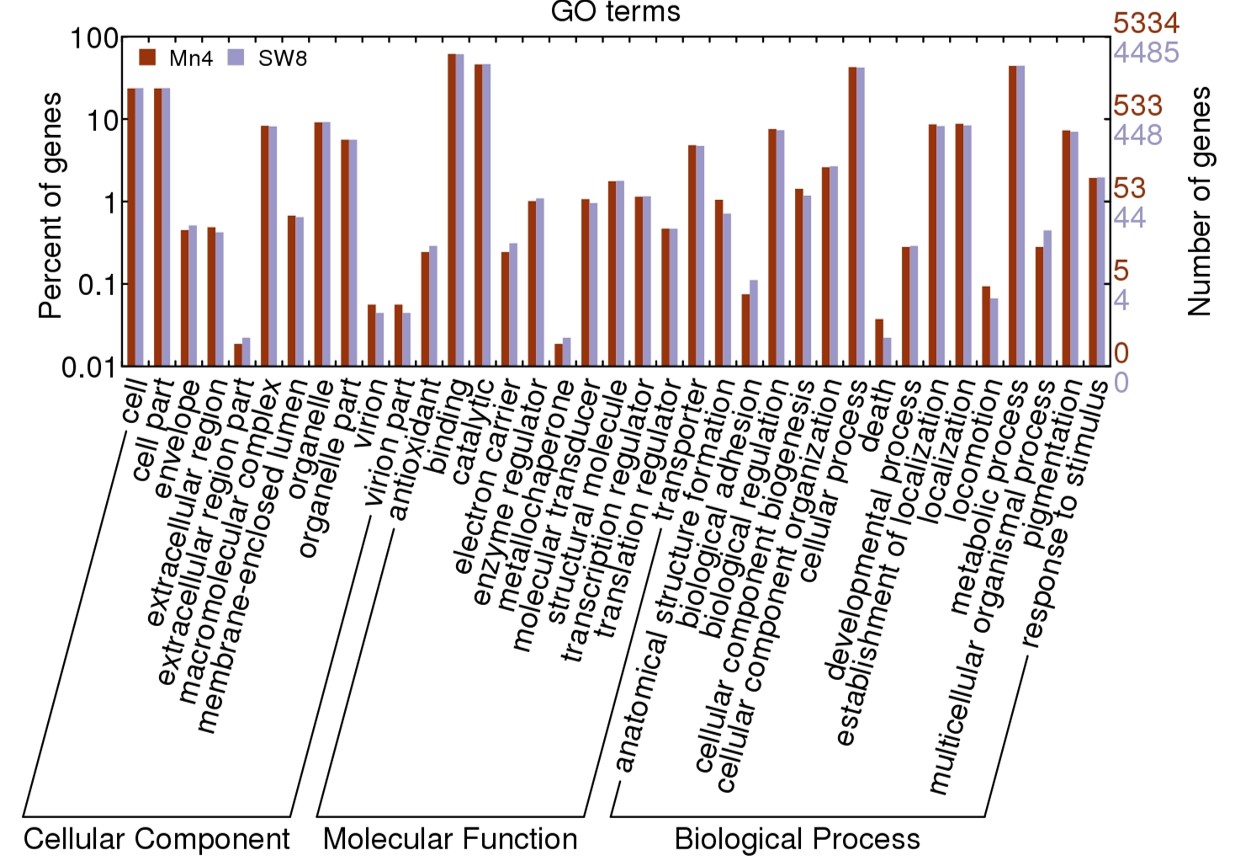

Supplement: Supplementary file 2 — Figure S1. Statistics for GO annotation of thraustochytrid strains Mn4 and SW8. (JPG 250 kb) [file 12864_2018_4904_MOESM2_ESM.jpg]

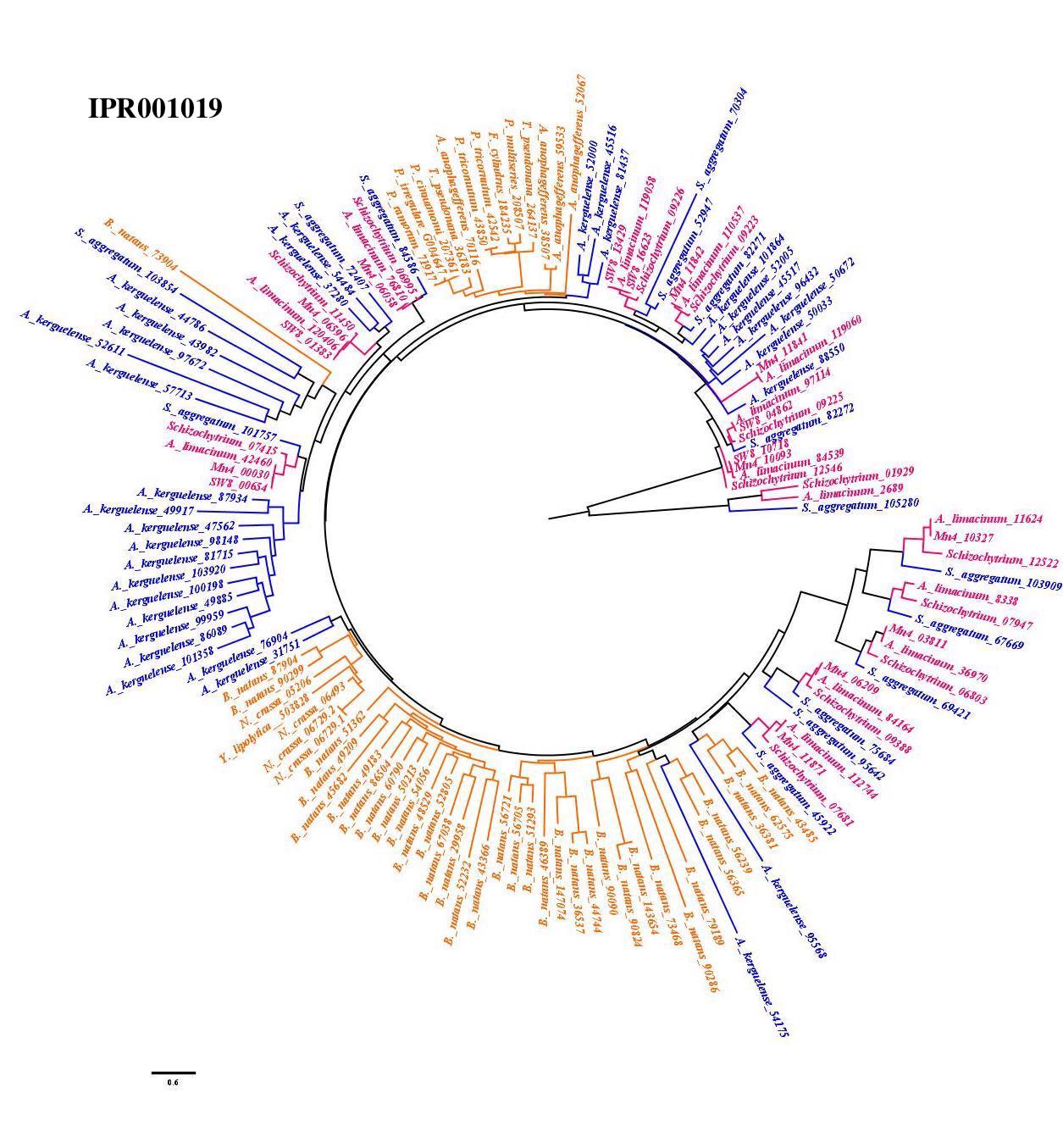

Supplement: Supplementary file 3 — Figure S2. Phylogenetic tree of all proteins containing IPR001019 domains. Total 18 species were assigned into 3 colored groups: pink for DHA-producing thraustochytrids, blue for non DHA-producing thraustochytrids and orange for 12 non thraustochytrids species. (JPG 217 kb) [file 12864_2018_4904_MOESM3_ESM.jpg]

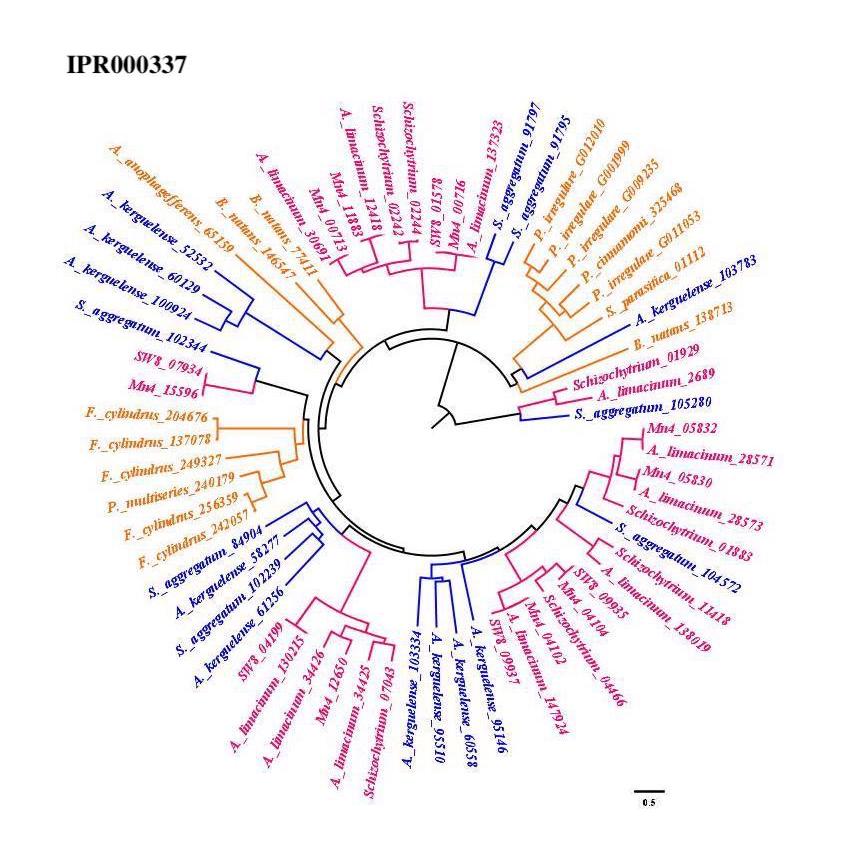

Supplement: Supplementary file 4 — Figure S3. Phylogenetic tree of all proteins containing IPR000337 domains. (JPG 103 kb) [file 12864_2018_4904_MOESM4_ESM.jpg]

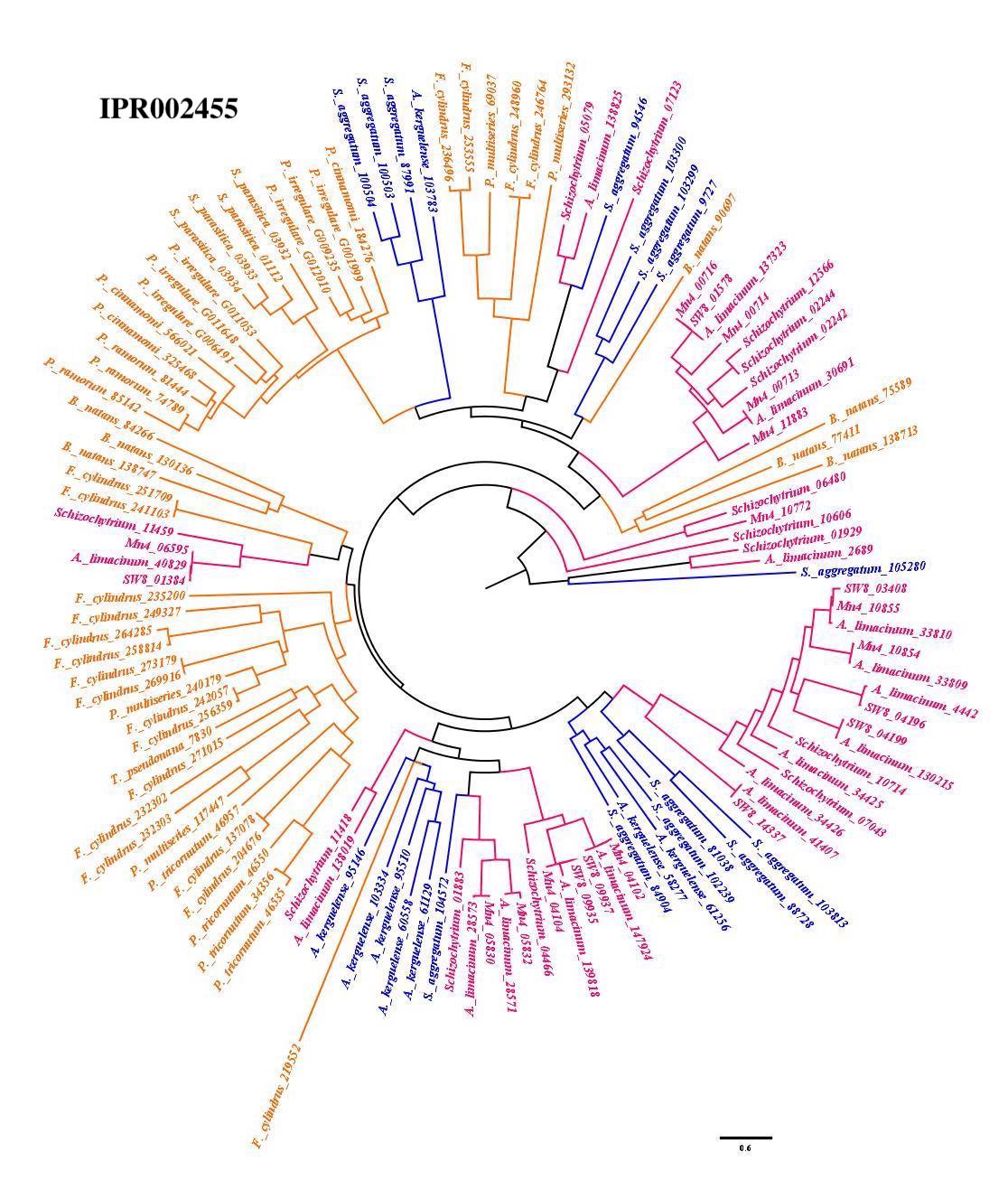

Supplement: Supplementary file 5 — Figure S4. Phylogenetic tree of all proteins containing IPR002455 domains. (JPG 215 kb) [file 12864_2018_4904_MOESM5_ESM.jpg]

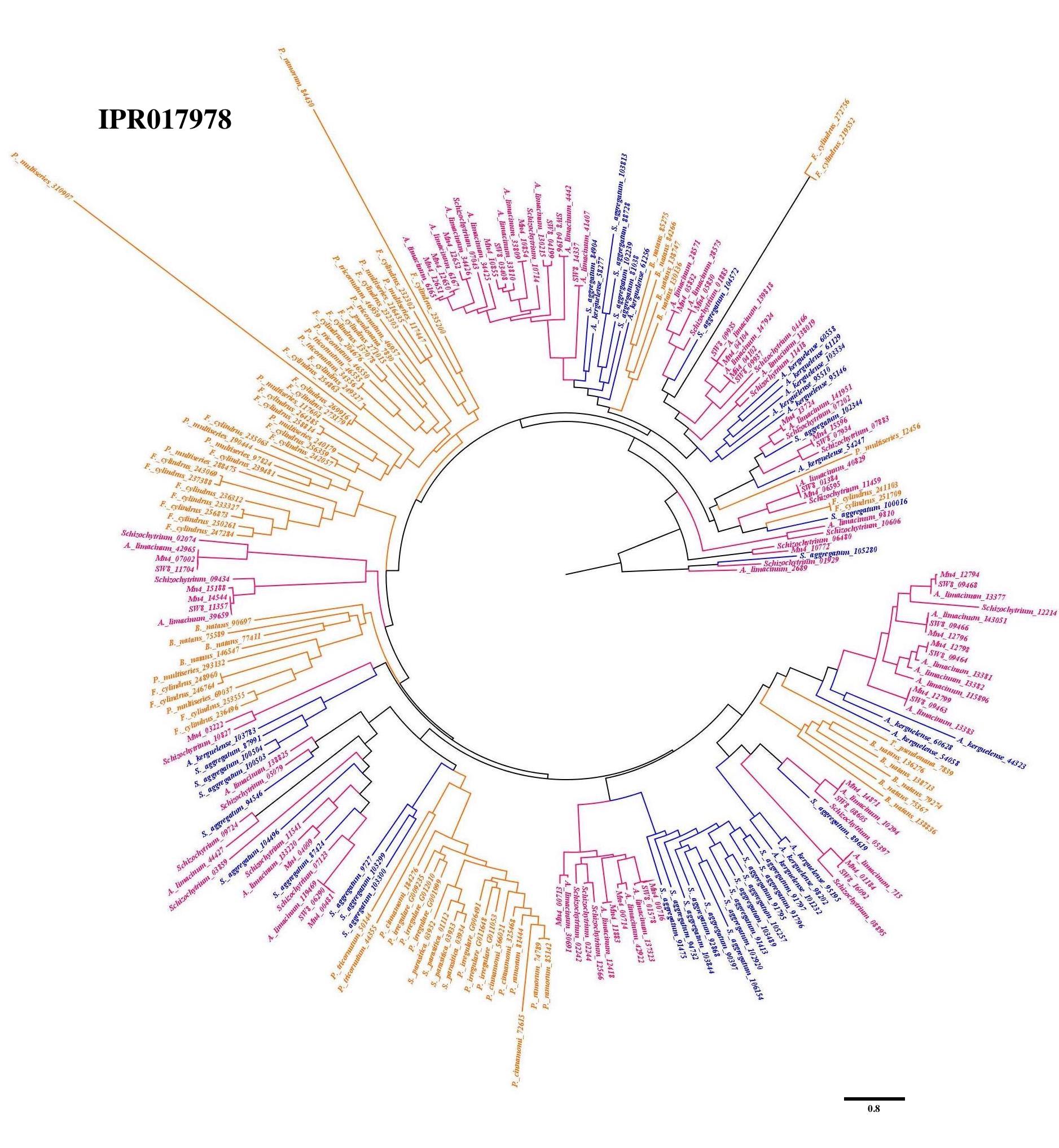

Supplement: Supplementary file 6 — Figure S5. Phylogenetic tree of all proteins containing IPR017978 domains. (JPG 406 kb) [file 12864_2018_4904_MOESM6_ESM.jpg]
